# Oral Health Knowledge, Attitudes and Behaviour of Parents and Caregivers of Preschool Children: Implications for Oral Health Promotion

**DOI:** 10.3290/j.ohpd.a43357

**Published:** 2020-07-04

**Authors:** Rahul S. Naidu, June H. Nunn

**Affiliations:** a Professor, School of Dentistry, The University of the West Indies, Trinidad and Tobago. Conception and design of the study, data acquisition, data analyses and interpretation, writing of the manuscript.; b Professor, School of Dental Sciences, Trinity College Dublin, Ireland. Conception and design of the study, data interpretation, writing and review of the manuscript.

**Keywords:** early childhood caries, parents, caregivers, oral health promotion

## Abstract

**Purpose:**

The aim of this study was to describe oral health knowledge, attitudes and behaviours of parents and caregivers of preschool children in order to inform an oral health promotion strategy.

**Materials and Methods:**

A sample of parents and caregivers of children attending nine randomly selected preschools in central Trinidad were invited to complete a self-administered questionnaire on early childhood oral health.

**Results:**

A total of 309 parents and caregivers participated: 88% were female, 74.4% were of Indian ethnicity, with 50.4% in manual employment, and 50.2% educated to secondary level. 59.1% felt a child’s first dental visit should be when all primary teeth are present. 64% had not taken their child for a dental visit. 81.6% rated their child’s oral health as good or better and 28% would want an asymptomatic, decayed primary tooth extracted rather than filled. Over 80% used fluoride toothpaste. 52.8% always supervised their child’s toothbrushing, and 44% claimed to be using the recommended pea-size amount. 26.2% reported having used a sweetened feeding bottle or infant feeder at night.

**Conclusion:**

Parents and caregivers of preschool children in this sample had reasonable oral health knowledge. However, despite generally positive attitudes towards preventive oral healthcare, confusion regarding dental attendance, supervised toothbrushing, fluoride use and sugar intake suggests that these items require particular emphasis in oral health promotion programmes aimed at improving early childhood oral health.

Poor oral health in early childhood is a serious public health problem.^25^ In particular, early childhood caries (ECC) and decayed teeth in children under 6 years of age can have negative effects on growth and development due to pain and discomfort, feeding and eating problems, reduced weight gain and poorer quality of life.^9,29^ The prevalence of ECC has been reported to range from 6% to 90%, with most developed countries in the lower end, and most developing countries in the middle to higher end of this range.^25^

Multilevel influences on children’s oral health at the individual, family and community levels have been proposed in the conceptual model of Fisher-Owens et al.^13^ Family level influences are mediated mainly through parents and caregivers with whom preschool children spend most of their time. During this period of primary socialisation, routine dietary and health behaviours are directly and indirectly influenced by the oral health knowledge, attitudes, beliefs and practices of parents and caregivers.^3^ Oral health knowledge and self-efficacy have been shown to influence oral health habits and routines in the home.^11^

Trinidad and Tobago is an English-speaking island in the southern Caribbean. National data on the oral health of primary school children has indicated a high prevalence of caries in the primary dentition.^24^ Much of this disease is likely to have started in the preschool years. Of concern was that parents and caregivers of preschool children attending a dental hospital clinic in Trinidad had inaccurate factual knowledge and low awareness of preventive care.^22^ A more recent, qualitative study found that although parents and caregivers of young children had generally positive attitudes to oral health, they reported encountering several barriers to accessing preventive care for their child with respect to maintaining healthy diets, good oral hygiene and regular dental attendance.^23^

The aim of this study was to describe oral health knowledge, attitudes and behaviours of parents and caregivers of children attending preschools in Trinidad, with a view to informing an oral health promotion strategy for early childhood.

## MATERIALS AND METHODS

The target population were children of preschool age in Trinidad. The accessible population were children attending preschools in the Caroni Education District of Central Trinidad, registered with the Ministry of Education.

Sample size was calculated using an estimated caries prevalence of 30% and a preschool population of 3800. A sample size of 250 was calculated based on a 6% level of precision. Assuming a 20% non-response rate, the target population size was 294. With an estimate of 30 children per preschool, this required 10 preschools in the sample. Preschools were recruited from a list of government/government-assisted and non-government preschools in the Caroni Education District registered with the Trinidad and Tobago Ministry of Education. Based on this list, there were 27 government and 57 non-government preschools in the District at the time of the survey. From these preschools, the final random sample resulted in three government/government-assisted and seven non-government-assisted preschools. One preschool was subsequently excluded as all children were under 3 years of age and thus did not fit the inclusion criteria. Data were collected over a 3-month period.

Ethical approval for the study protocol was obtained from the University of the West Indies Faculty of Medical Sciences Research Ethics Committee

Letters of request to participate in the survey were sent to each preschool in the sample, addressed to the head teachers/administrators. For preschools that agreed to be in the study, packages were sent to each school head teacher/administrator that included letters for individual parents and caregivers, inviting them to take part in the study and complete a self-administered questionnaire. The questionnaire was developed from an instrument previously used in Trinidad^22^ and the USA.^10^

Questionnaire variables included: parent/caregiver age, sex, ethnicity; occupation of head of household; level of education; oral health rating; dental attendance; oral health knowledge; beliefs and practices in relation to young children; health literacy; oral health self-efficacy; stress and social support; parental rating of child oral health; child dental attendance.

## RESULTS

For the 340 children enrolled in the nine preschools in the final sample, 309 parents and caregivers responded to the invitation to participate in the survey and completed the dental health questionnaire (response rate 91%).

### Sociodemographics

The majority of parents and caregivers were in the 25–44 age range. A total of 271 (88%) were female and 38 (12%) male (Table 1). The largest ethnic group were those of Indian descent (74.4%) followed by Mixed (13.3%) and African (11.3%). The participants’ children were in the following age ranges: 3 years (38.5%), 4 years (51.5%) and 5 years (10%). Half of the participants (50.4%) had a manual-working head of household and a third (33.3%) were from non-manual-working households. The majority of participants (50.2%) were educated to at least secondary level (Table 1).

**Table 1:** Sociodemographic information for all respondents (n = 309)

	%
**Age group**	
18–24	5.5
25–34	59.2
35–44	30.7
45–54	2.6
55–64	0.7
65+	0.3
Missing	1.0
**Ethnic group**	
African	11.3
Indian	74.4
Mixed	13.3
White	0.3
Other	0.7
**Occupation**	
Professional	3.6
Managerial/lower professional	15.5
Non-manual	14.2
Manual – skilled	34.6
Manual – semi-skilled	1.9
Manual – unskilled	13.9
Housewife/unemployed	4.9
Retired/old-age pensioner	2.3
Missing	9.1
**Education**	
None	0.3
Primary	9.4
Secondary	50.2
Technical college	17.5
University	15.9
Other	4.9
Missing	1.9

### Oral Health Knowledge

Most participants were aware of the causes of caries and the role of fluoride. Almost 15% disagreed with or did not know if baby teeth should be brushed as soon as they come into the mouth. Almost 10% did not know if putting a child to bed with juice could cause cavities and 31% either did not know or agreed that ‘tooth decay ran in families’ (Table 2).

**Table 2:** Parent/caregiver knowledge and attitudes towards children’s oral health and causes of caries (n = 309)

Statement	Agree %	Disagree %	Don’t know %	Missing %	Total %
(a) Parents should start cleaning their child’s teeth as soon as the first baby tooth comes out	83.8	9.1	5.5	1.6	100
(b) Germs in the mouth can cause tooth decay	97.7	0.3	1.3	0.3	100
(c) Fluoride helps to prevent tooth decay	87.1	1.9	5.5	5.5	100
(d) Putting a child to bed with a bottle containing juice can cause cavities in teeth	87.7	1.9	9.4	1.0	100
(e) Tooth decay runs in families	12.9	61.5	17.8	7.8	100

### The First Dental Visit, Oral Health Rating, Treatment for a Decayed Tooth

Most participants (59.1%) felt that the first dental visit for a child should be when all the primary teeth have erupted. A total of 24% thought this should be when they get their first adult teeth. Over a fifth (22%) were unsure of when the first visit should be (Table 3). Most participants (81.6%) rated their child’s oral health as good or better and 17% as fair or poor (Table 3). Almost two-thirds of participants’ children (64%) had not had a dental visit. Ten per cent (10%) of participants had experienced difficulty in finding dental care for their child. Over a third of participants (37%) were unsure of what to do about a symptomless decayed primary tooth. Over a quarter (28%) of respondents would want the tooth taken out rather than filled (22%). Thirteen per cent (13%) would want no treatment (Table 3).

**Table 3:** Parent/caregiver knowledge of the first dental visit, rating of their child’s oral health and treatment preferences for an asymptomatic carious primary tooth (n = 309)

Time for the first dental visit	%
When they get their first tooth	5.8
When they have all their baby teeth	59.1
When they get their first adult tooth	7.8
Only when they get pain or have a problem	3.6
Not sure	22.0
Missing	1.0
Child’s oral health rating	
Excellent	18.8
Very good	29.8
Good	33.0
Fair	12.3
Poor	4.9
Missing	1.3
Preferred treatment for a decayed baby tooth that was not causing pain	
Nothing	13.3
Have it filled	21.7
Take it out	27.8
Not sure	36.6
Missing	0.7

### Toothbrushing

The majority of participants reported that their child had their teeth brushed at least twice a day and just under a third of the sample at least once a day (Table 4). Most participants (63%) helped to brush their child’s teeth from in front of the child and 15% from one side. Most participants (88%) used a small toothbrush for their child and 11% a medium brush size (Table 4). Nearly all participants let their child use toothpaste when brushing. Over half of the participants (56%) reported the use of a quantity of toothpaste that would cover half or all of the brush head of their child’s toothbrush. Forty-four per cent of participants were using a pea-size amount (Table 4).

**Table 4:** Parent/caregiver toothbrushing behaviours, type of toothbrush used and quantity of toothpaste (n = 309)

How often does your child brush their teeth	%
Once a day	32.0
Twice a day	61.2
More than twice	5.8
Missing	5.8
How often do you help your child brush their teeth	
Sometimes	46.3
All the time	52.8
Never	1.0
Position when supervising brushing	
In front	62.5
Behind	14.2
At one side	15.5
Missing	7.8
Size of toothbrush	
Small	88.4
Medium	11.3
Large	0
Missing	0.3
Amount of toothpaste	
Enough to cover the whole brush head	32.0
Enough to cover half the brush head	23.6
Pea-size	43.7
Don’t know	0.7

### Fluoride Use

Over 80% participants were using fluoride toothpaste for their child (Table 5). Most participants had not used any other fluoride vehicles for their child (58.9%). Less than a tenth of participants reported fluoride varnish having been applied to their child’s teeth (Table 5).

**Table 5:** Parent/caregiver use of fluoride vehicles, history of breastfeeding and night-time bottle feeding (n = 309)

Child’s toothpaste contains fluoride	%
Yes	88.4
No	3.6
Don’t know	4.9
Missing	3.2
Other fluoride vehicles	
Fluoride tablets/drops	0.3
Fluoride varnish or gel applied by a dentist or dental nurse	9.4
Fluoride mouthwash	31.4
No other fluoride	58.9
Age stopped breastfeeding	
<3 month	5.2
3–6 months	28.2
6–12 months	19.7
>12 months	22.0
Not breastfed/missing	24.9
Used a sweetened baby bottle/ infant feeder at night	
Yes	26.2
No	71.2
Missing	2.6

### Breastfeeding and Dietary Practices

- Over three-quarters of participants reported that their child was breastfed.
- A third of participants breastfed their child up to 6 months of age and one-fifth up to a year.
- Over a fifth of children (22%) were breastfed past 1 year of age (Table 5).
- Eight children (3%) with a mean age 3.1 years were still being breastfed at the time of the study.
- Over a quarter (26.2%) reported having given their child a sweetened baby bottle or infant feeder at night (Table 5).
- Over half of participants (58%) gave sweet drinks to their child twice a day or more, while a third (33%) reported giving sweet snacks twice or more than twice a day.
- A fifth of respondents reported that their child ate fruit more than twice day (Table 6).

**Table 6:** Child frequency of intake of sweet drinks, snacks and fruits (n = 309)

Food/drink	Never %	Rarely %	Once a day %	Twice a day %	More than twice a day%	Missing %	Total %
Sweet drinks	0	15.2	24.9	27.8	30.1	1.9	100
Sweet snacks	1.0	23.3	40.5	17.1	16.2	1.9	100
Fruits	0.7	6.5	45.6	25.2	20.7	1.0	100

### Parent/Caregiver Dental Attendance, Oral Health Rating, Stress and Oral Health Literacy

Thirty per cent (30%) of participants attended the dentist once a year. Over a quarter (26%) reported attending for dental care every 6 months and 14% only attended when in pain (Table 7), while 17% reported having difficulty in finding dental care for themselves.

**Table 7:** Parent/caregiver dental attendance, dental health self-rating and experience with finding dental care (n = 309)

Frequency of dental visits	%
Never been	16.5
Only when in pain	13.9
Every 6 months	25.9
Once a year	30.1
Other	11.1
Missing	1.6
Dental health self-rating	
Excellent	7.1
Very good	22.7
Good	40.9
Fair	21.0
Poor	6.8
Missing	1.6
Difficulty in finding dental care	
Yes	17.8
No	79.0
Missing	3.2

Most (71%) participants rated that their dental health as good to excellent and over a quarter (28%) rated it as fair to poor (Table 7). But over half (56%) of participants reported that their child made too many demands on them, occasionally to all the time (4.2%). Eighteen per cent (18%) of participants reported needing help with reading health information, ‘occasionally’ to ‘all the time’ (Table 8).

**Table 8:** Parent/caregiver stress and oral health literacy (n = 309)

How often do you feel your child is making too many demands on you?	%
All the time	3.9
Often	5.5
Sometimes	22.7
Occasionally	23.6
Never	40.8
Missing	3.6
How often do you need help to read information from leaflets or written material from a doctor or pharmacy?	
All the time	1.3
Often	1.3
Sometimes	8.1
Occasionally	7.1
Never	81.9
Missing	0.3


### Effect of Socioeconomic Status

Bivariate analysis found no associations between occupation of head of household/level of education and oral health knowledge, attitudes and behaviour, with respect to their child.

Associations were found for these variables with respect to the participants’ own oral health. A greater proportion of participants from households in manual employment had difficulty in finding dental care for themselves (chi-squared p < 0.05). In addition, a greater proportion of participants from manual households and those with secondary school as their highest level of education, visited the dentist less frequently than participants who were from non-manual households or had been educated above secondary level (chi-squared p < 0.01).

A greater proportion of participants with secondary school as their highest level of education rated their oral health as fair or poor, compared to those educated above secondary school level (chi-squared p < 0.01)

## DISCUSSION

Parents and caregivers of preschool children in this sample had fair oral health knowledge. However, despite generally positive attitudes towards preventive oral healthcare, confusion regarding dental attendance, supervised toothbrushing, fluoride use and sugar intake suggest these items require particular emphasis in oral health promotion programmes aimed at improving early childhood oral health.

A good response rate (91%) was achieved from the random sample. As would be expected, the majority of respondents were female since mothers, or other female family members, are often the main caregivers in the family. Half of the respondents were in the 25- to 34-year age range, indicating a relatively young group, which is consistent with them having children at the preschool stage. The majority of participants were from families with a head of household in manual work and over half not educated beyond secondary school level, thus the sample was predominantly made up of families from lower socioeconomic status.

Almost three-quarters of the sample were of Indian descent, which differs from the national demographic profile of Trinidad and Tobago, where African and Indian ethnicity are in similar proportions.^7^ This distribution reflects the ethnic composition of the region where the study took place. The Caroni region is located in the central part of the island of Trinidad and is historically the hub of the sugar cane industry. Indian indentured labourers arrived during British colonial rule to work in the plantations and sugar mills.^4^ Despite its agricultural past, the area is presently a mixture of urban and rural communities.

It has been reported that populations working in the sugar industry have a higher prevalence of dental caries than the general population.^14,28^ In the Caribbean, a common taste preference for highly sweetened foods and drinks may be a legacy of the sugar industry. For instance, along with the general popularity of confectionary and highly sweetened drinks, raw sugar cane is still available as a sweet snack, sold in local convenience stores and groceries.

Of concern is that sweetened drinks and snacks were given frequently to the majority of children in this sample, suggesting that conditioning to a high sugar intake may occur in early childhood. Frequent intake of sweetened drinks and confectionary that contain free sugars are established risk factors for ECC.^8^ Sweetened beverages should be confined to mealtimes. In tropical climates, including the Caribbean, children need regular hydration, therefore parents and caregivers must be advised on alternative drinks such as water, milk or unsweetened fruit drinks as dentally safe options. However, challenges for health promotion in this respect are highlighted by recent research reporting daily consumption of sugar sweetened beverages (SSBs) from around the world, which found the highest values in the Caribbean region.^30^ The report also showed that within the Caribbean region, Trinidad and Tobago had the highest daily SSB consumption, at over twice the global average.

Following a worldwide consultation on public health solutions to ECC, the World Health Organization recently provided recommendations for prevention and management of ECC in low- to middle-income countries.^26^ The consultation recommended that preventive approaches for ECC should consider sociobehavioural risk factors, including children’s diet and infant feeding practices and policies to reduce sugar consumption, such as taxation for SSBs and highly sweetened unhealthy foods.

Over a tenth of respondents agreed with the statement ‘tooth decay runs in families’ and almost a quarter did not know or did not agree with this statement. Some of these parents and caregivers may therefore believe that caries in the primary teeth of very young children is inevitable, suggestive of a fatalistic view of their child’s oral health. People with fatalistic health beliefs have lower perceived need for care and lower utilisation of health services.^15^ In a US study of low-income African-American families with preschool children, the authors found that higher oral health knowledge was protective against ECC but caregivers espousing fatalistic beliefs tripled the odds of their children having ECC.^11^

Oral health knowledge and attitudes, and their effect on oral health behaviours, are also mediated by social and cultural beliefs and norms within a community.^27^ In the present study, there were generally positive attitudes about the care of a carious primary teeth, as measured by the response to the question ‘if your child had decay in a baby tooth that was not causing pain, what treatment would you want?’ However, there was some ambivalence, with over a third of respondents unsure.

The majority of respondents indicated that their preschool-aged child had not visited a dentist. Best-practice guidelines recommend that a child be taken for a dental check-up by 1 year of age, ideally within 6 months of eruption of the first tooth.^1^ Early dental attendance can also enable the delivery of ‘anticipatory guidance’ defined as ‘the process of providing practical, developmentally appropriate health information to caregivers, in anticipation of significant emotional, physiological milestones’.^5^ Early attendance may enable use of topical fluoride therapy, which has been shown to be effective.^19^

In their systematic review, Hooley et al reported that supervised brushing was associated with less caries experience.^16^ In the present study, most children were reported to have their teeth brushed at least once a day, with over two-thirds brushing twice a day or more. Nearly all the respondents reported helping their child brush some or all of the time. This is encouraging, as although some preschool children are happy to brush, they lack the manual dexterity to do so effectively and supervised toothbrushing or assisting with brushing is recommended until children can do this effectively, by around the age of 7 years.^18^

Psychosocial factors may influence engagement in, and maintenance of, health-promoting behaviour such as supervised toothbrushing and, in particular, a mother’s level of self-efficacy.^11^ Self-efficacy has been defined as an individual’s belief in their ability to carry out and succeed at a specific task.^20^ Therefore, although improving oral health knowledge may not directly influence oral health behaviour, it may exert an indirect influence through developing self-efficacy.

Over half the respondents in the present study reported that their child sometimes made too many demands on them, suggesting that interventions aimed at changing oral health behaviour should be sensitive to the wider concerns of parents and caregivers and the overall demands of child rearing.

Nearly all respondents in this study reported using toothpaste that contained fluoride. Of concern is that over half were using enough toothpaste to cover half or all the brush head, rather than a pea-sized amount. Overingestion of toothpaste in preschool children can occur due to their inability to spit out effectively after brushing. Use of the correct amount of toothpaste is therefore important to reduce the risk of children developing enamel opacities in their permanent teeth as a consequence of ingestion of fluoride. Current guidance recommends twice-daily brushing with a small-headed soft toothbrush and a smear of toothpaste (0.1 mg F) for children under 3 years of age and a pea-size amount (0.2 mg F) for children age 3 to 6 years.^26^

Three-quarters of the respondents reported that their child was breastfed and just over a fifth continued this practice past 1 year. This is consistent with national data that reports that a fifth of mothers in Trinidad and Tobago were breastfeeding up to 24 months.^21^ UNICEF guidelines on breastfeeding are endorsed in Trinidad and Tobago and widely disseminated. These recommendations include ‘exclusive breastfeeding for the first 6 months and following introduction of complementary foods at 6 months to continue breastfeeding for at least two years’. Dental health advice must therefore be developed with sensitivity to the wider health promotion agenda. For instance, in their position statement on infant feeding, the British Society for Paediatric Dentistry state that mothers should work closely with all their health practitioners to minimise the potential risk for dental caries.^6^

 In the present study, over one-quarter of caregivers reported that they gave their child a sweetened baby bottle or infant feeder at night-time. There is evidence from case reports, cross-sectional and longitudinal studies using multivariate analyses to show that night-time feeding using a sweetened baby bottle or comforter increases the risk for ECC, with the maxillary incisors being most vulnerable.^8^ Giving a child a bottle to help them sleep or comfort may be a cultural norm for many caregivers; therefore, culturally sensitive approaches are required when developing oral health promotion initiatives.

Oral health literacy is ‘the degree to which individuals have the capacity to obtain, process and understand basic oral health information and services needed to make appropriate health decisions’,^17^ which is similar to findings in Australia.^2^ The majority of respondents in this study reported never having difficulty reading health information, however, the availability and relevance of the information must also be considered when developing health education resources for the public.

## CONCLUSIONS

### Recommendations

Consistent with international guidelines the findings from this study support the following recommendations for an oral health promotion approach for ECC:

***Dental attendance and caries risk assessment***
  - Child to register at a dental clinic ideally by age 1.
  - Establish a dental routine for the child.
  - Clinical caries risk assessment based on a standardised assessment tool and criteria.
***Toothbrushing and instruction***
  - Child to have supervised toothbrushing at least twice a day (ideally last thing at night and one other occasion).
  - Use a pea-size amount of toothpaste (1350–1500 ppm fluoride).
***Dietary advice***
  - Dental professionals should advise on dentally safe breastfeeding, taking account of local policy on breastfeeding from mother and child clinic/community nurses.
  - Parents and caregivers advised not to put the child to bed with a feeding bottle containing sweetened liquids.
  - Parents and caregivers should be advised to minimise frequency of foods containing added sugars (restrict to mealtimes).
  - Parents and caregivers should be advised against using drinks containing added sugars in feeding bottles.
  - Recommend low sugar snacks (eg, cheese, fruits).
  - Snacks containing sugar substitutes preferable to those containing added sugars.
***Community-based prevention***
  - Develop and implement supervised toothbrushing programmes in preschools and day-care settings.
  - Promote brushing with toothpaste containing fluoride at 1000 ppm+.
  - Create supportive environments for healthy oral health behaviour in preschools (lunchbox policies, breaktime snacks, tuck shop policies on availability of non-sugary snacks).
  - Implement programmes on oral health for pregnant women, following on to the peri/post-natal period.
  - Involvement of non-dental health professionals in oral health promotion for families with young children (eg, general practitioners, paediatricians, nurses, preschool teachers, community workers and lay educators should be involved).
  - Introduce brief counselling techniques (eg, motivational interviewing) to aid delivery of dental health advice and facilitate behaviour change.

### Limitations

The possibility of selection bias must be considered in this study. Not all children in this age group attend preschools or day-care facilities and their sociodemographic/behavioural characteristics may have differed from those children sampled through preschool enrolment.

The use of self-reported questionnaires for a period in the preceding 6 months can be affected by respondent recall and some participants may have been influenced by the social desirability for the ‘right’ responses to questions such as those on toothbrushing and dental attendance (ie, a tendency to present one’s self in the best possible light).^12^
